# Insurance-based disparities in breast cancer treatment pathways in a universal healthcare system: a qualitative study

**DOI:** 10.1186/s12913-023-09108-0

**Published:** 2023-02-03

**Authors:** Zeynep Kesici, Volkan Yilmaz

**Affiliations:** 1 Social Policy Forum Research Centre, Boğaziçi University, Istanbul, Türkiye; 2 School of Law and Government, Dublin City University, Dublin, Ireland

**Keywords:** Breast cancer, Disparities in healthcare, Health insurance, Healthcare systems, Treatment pathways, Universal health coverage

## Abstract

**Background:**

The influence of healthcare system factors on treatment pathways for breast cancer has been studied extensively in lower-middle-income countries (LMICs), but in upper-middle-income countries (UMICs), this area is understudied. This article focuses on the experiences of breast cancer patients in Türkiye, a UMIC with a universal healthcare system. It explores variations in treatment pathways based on the type of health insurance provider (private or state).

**Methods:**

The study uses an exploratory qualitative method based on in-depth interviews with 12 breast cancer patients. The inclusion criteria were Turkish nationality, female gender, and having received treatment from a private hospital within one year of the interview. A purposeful sampling strategy was employed to recruit patients who had either social health insurance only or who had private health insurance in addition to their social health insurance. A two-stage thematic analysis of the interview data was conducted. First, we examined whether the type of insurance provider makes a difference in treatment pathways; we then identified healthcare system factors that explain these differences.

**Results:**

The study revealed two distinct pathways to treatment. These differ in terms of financial protection, service coverage, and patients’ sense of equity. Patients with private insurance reported easy access to timely and comprehensive treatment. Those without, however, had to navigate complicated routes to treatment; they generally had to resort to seeking treatment from more than one hospital. We found two healthcare system factors that explained the differences: a failure to fully enforce the mandates of the state’s social health insurance in the private hospital sector and growing reliance on private insurance to gain access to essential services.

**Conclusions:**

Based on data from the Turkish case, we conclude that healthcare system factors are indeed influential in shaping treatment pathways for breast cancer in UMICs with universal healthcare. These factors include a failure to fully enforce the mandates of the state’s social health insurance programme in the private hospital sector and a growing reliance on private insurance to gain access to essential services. We note that this contrasts dramatically with the situation in LMICs, where the main factors are low-quality care and shortages of medical staff, medicines, and technologies.

## Background

### Breast cancer treatment pathways

Breast cancer is an exceptionally prevalent chronic illness among women worldwide. Although overall mortality has decreased since the 1980s thanks to preventive and therapeutic interventions, the availability of diagnosis and treatment services varies substantially across countries. Clinical guidelines for treatment are, for the most part, comparable across countries and providers; treatment pathways, however, often vary, even within a single country. Not all differences in treatment pathways imply a disparity in access to treatment and patient outcomes, but some do point in that direction and therefore deserve further empirical investigation. This is particularly the case if the difference implies a delay in treatment, which is found to increase mortality [1].

Patient pathways to treatment represent a relatively new agenda in research on healthcare systems and services. The term ‘treatment pathway’ refers to how patients navigate the healthcare system during treatment for their illness [2]. ‘Treatment pathway’ is used interchangeably in the literature with ‘patient pathway,’ ‘patient journey’ and ‘cancer journey’ [3]. The present study adopts Richter and Schlieter’s view that patient pathways should not be reduced to a set of standardised procedures; rather, they should be characterized as an ‘unplanned journey’ [2, p. 993]. Similar to several previous studies [4–6], this article takes a bottom-up approach to examining patient pathways, that is, by starting with individual patient experiences and assembling them into specific pathway patterns.

Research into healthcare system influences on breast cancer treatment pathways is growing, but it focuses primarily on lower and middle-income countries (LMICs) [7–9]. A systematic review of studies on sub-Saharan African countries [8] identified several healthcare system factors such as diagnostic process failures, cost, and provider attitudes, all of which can erect barriers to access that result in a delay in the diagnosis and treatment of breast cancer patients. ‘Provider attitude’ in that review refers specifically to the pervasive prevalence of corruption, bribery, and nepotism within the medical staff. Another systematic review [7] —this one on healthcare system barriers to breast cancer diagnosis and treatment in LMICs in Asia—arrives at conclusions similar to those of Gbenonsi et al.’s review [8]. This review on Asia reveals that the most important barriers are low-quality healthcare, shortages of health personnel, medicines and medical technologies, and a lack of insurance, which has implications for out-of-pocket payments.

Studies on how healthcare system factors influence breast cancer treatment pathways in upper-middle-income countries (UMICs) and high-income countries are limited in number. A rare study in Thailand [10], a UMIC, finds that it is patient beliefs, not healthcare system factors, that explain delays in cancer treatment. In a Canadian study [11], the researchers observed a negative relationship between physical distance from radiotherapy centres and cancer outcomes. Studies in the United States [12, 13] mention underinsurance as a healthcare system factor that impedes access to breast cancer diagnosis and treatment. Finally, a systematic review of financial distress reported by cancer patients in universal healthcare systems [14, p. 14] concludes that ‘the design of healthcare and social security systems might also shape the extent of not only objective financial burden but also protection of vulnerable cancer patients at risk of financial distress.’

To explore the influence of healthcare system factors on breast cancer treatment pathways, we combined a bottom-up approach to patient pathways with a context-sensitive approach. This is important, as patient experiences do not occur in a social and historical vacuum. We interpreted individual patient experiences in the light of the healthcare system context. This helped us ascertain whether differences in treatment pathways were caused by healthcare system factors and if, in turn, these differences created inequalities in healthcare.

Breast cancer treatment in Türkiye offers an interesting case to examine the role of healthcare system factors in shaping these treatment pathways, first of all because breast cancer is highly prevalent [15]; its incidence reached 56.6 per 100,000 in 2020 [16, 17] and continues to rise. Late diagnosis is also frequent [16]; Turkish breast cancer is often diagnosed only when the patient presents to the hospital with symptoms. Importantly, Türkiye is a UMIC with a universal healthcare system where the state’s social health insurance (SHI) pays for all breast cancer treatment, whether it is from a public or private hospital.

Theoretically, the Turkish healthcare system is a publicly-funded internal market, as defined elsewhere [18] that brings together SHI for all with fully comprehensive coverage and gives patients the right to choose their treatment provider. Patients in Türkiye are charged a flat fee (a co-payment) for outpatient visits and prescribed medications, and they pay variable rates of co-insurance for outpatient visits to private hospitals only [19]. Co-payments are modest, but the charges for co-insurance can be considerable. Although co-insurance is required for most private services, breast cancer treatment is exempt; it is completely free of charge at the point of service [20]. Türkiye has witnessed an upsurge in the number of its citizens who hold private health insurance (PHI) in the last decade, which is an intriguing trend in a universal healthcare system, up from less than a million in 2010 to more than 6.5 million (out of a population of almost 85 million) by the end of 2021 [21].

In the context of the Turkish healthcare system described above, we would not have expected to observe insurance-based differences in breast cancer treatment pathways. Two assumptions underlie our expectation: (1) the SHI should be providing the same comprehensive coverage to all patients, and (2) breast cancer treatment is exempt from co-insurance payments. Nevertheless, we observed considerable insurance-based differences in treatment pathways for breast cancer. We argue that these differences can be explained by factors that are specific to the Turkish healthcare system.

Finally, this study on Türkiye also speaks to the literature on patient choice and equity in access in UMICs with universal healthcare systems. Data is also scarce on how patients view and experience choice in healthcare in UMICs that have undertaken patient choice reforms (Thailand and Türkiye, for instance), and most existing studies have been conducted in the Global North [22]. In the countries of the Global North, scholars have observed a disharmony between choice and equity in healthcare systems [23–26]. Whether a similar tension exists between choice and equity in UMICs with universal healthcare systems is still an open question.

## Methods

### Study design and case selection

We used an exploratory qualitative methodology to obtain insights into differences in breast cancer treatment pathways and healthcare system factors that influence them. To gain a nuanced and detailed understanding, we used a bottom-up approach, starting with individual experiences and then working upward to understand the larger systems and structures in which these individual experiences are situated. The bottom-up approach made it possible to detect discrepancies between statutory entitlements to healthcare and the actual experiences of patients. It also gave us insights into the complex interplay between entitlements and the functioning of the healthcare system and helped us identify areas that need improvement.

This study draws on semi-structured in-depth interviews with 12 breast cancer patients in Istanbul, Türkiye. Istanbul was selected as the site for this study because it has more private hospitals than other cities in the country and a greater number of PHI policyholders [21]. We investigated whether treatment pathways differ for breast cancer patients because of the type of insurance and whether and to what extent the statutory right to free breast cancer diagnosis and treatment is respected in the Turkish private-hospital sector.

### Data collection and analysis

The data for this study were collected in July and August 2021 using a purposeful sampling strategy to recruit an equal number of breast cancer patients with PHI and SHI-only (6 per group). The main inclusion criterion for participation was Turkish citizenship, female gender, and having received a breast cancer diagnosis and treatment in a private hospital within one year of the interview. Considering that Turkish breast cancer patients have become more open to discussing their experiences in the last decade [27], we employed a self-selection strategy as the initial recruitment method [28]. This involved contacting various cancer patient organizations in Istanbul and asking them to spread the call for participants to their members and followers. Individuals who were interested in participating were asked to contact us directly via email or telephone. Of the first seven potential participants who contacted us, five agreed to participate, and two declined after receiving more information about the interview procedures.

The strict health and safety measures that were in effect because of the COVID-19 pandemic at the time made it difficult to reach the target population for this study, so we then employed a snowball sampling approach, where we asked the five participants we had already interviewed to refer us to potential interviewees in their social circles. This snowball sampling approach yielded seven additional interviews. This strategy not only allowed us to reach a larger number of participants but also facilitated better communication and trust-building between the researchers and the new participants, which was particularly important given the potentially delicate nature of the topic being studied (as discussed in [29]). After the interviews, we did iterative readings of the data to identify common patterns and emerging points of interest. We found that the data had reached a point of saturation and no further new insights were gained, which led us to conclude that the final sample size of 12 participants was sufficient.

The final sample included 12 breast cancer patients who had received treatment in private hospitals in Istanbul within the past year. Six had duplicate coverage (both SHI and PHI), while the remaining six had only SHI. The median age was 45. Eight participants were employed, and four were retired. Amongst the PHI policyholders, three had purchased their policies individually, while others had policies that were provided by their employers. The participants had diverse educational backgrounds, but all were at least high school graduates. All personal data were meticulously anonymised during the transcription process. An identification tag was assigned to each participant to protect her identity.

The interviews lasted an average of one hour. Three were conducted in person at a public outdoor location chosen by the interviewee, and nine were held online via teleconferencing. The participants were asked about their experiences navigating the Turkish healthcare system with regard to their breast cancer diagnosis and treatment. The questions and prompts were structured to focus on the patients' experiences of accessing, receiving, and continuing treatment, rather than their experiences with the illness itself. The interviews, conducted in Turkish, were audio-recorded, transcribed verbatim, and analysed by the authors, who then translated selected relevant direct quotations into English for the purposes of this article.

To ensure an in-depth understanding of patient experiences, we employed an exploratory thematic analysis approach described by Braun and Clarke [30]. This approach was particularly well-suited to our research goals because it allowed us to explore the complex and nuanced experiences of our participants in a flexible and open-ended manner. To facilitate the organization and analysis of our data, we used NVivo 12 software. In the first round of systematic analysis, we used a blended approach to coding. For the first two themes (financial protection and service coverage), we decided to borrow from the literature on universal health coverage. We developed the third theme (a sense of equitable treatment) inductively from the data. To ensure the validity and reliability of our analysis, we engaged in regular discussions, carefully considering any inconsistencies or ambiguities until a consensus was reached on all three themes. In the second round of analysis, we examined the relationships between these three themes and sought to identify healthcare system factors that might explain any observed inequalities in access to treatment based on insurance coverage.

## Results

We observed that breast cancer treatment pathways diverge according to the type of health insurance. The pathways of PHI policyholders experienced easy and timely access to standard treatment, most often from a single private provider. This contrasts with the experience of patients who had only SHI; their pathways to diagnosis and treatment tended to be complicated, and they generally accessed their treatment from multiple providers. The following section presents differences in patient experiences in areas that account for the different pathways to treatment—financial protection, service coverage, and a sense of equitable treatment.

### Financial protection

The treatment pathways of SHI and PHI policyholders vary in terms of the amount of financial protection these groups enjoy in practice. Our analysis indicates that PHI policyholders enjoy a high level of financial protection, but patients with only SHI are obliged to pay considerable amounts out-of-pocket for their treatment in private hospitals and often suffer financial hardship and distress as a result. Recall that all cancer patients are legally entitled to standard cancer treatment free of charge in all hospitals, regardless of insurance type. Our study reveals, however, that only those insured by PHI did not have to consider the financial aspect of their treatment.

*‘I didn’t pay anything for my chemotherapy, my PHI covered it 100%. I didn’t pay a penny. If I had gone with the general health insurance [referring to SHI], I would have had to pay a difference [referring to co-insurance] of 600 Turkish Liras (TL) at K hospital for each chemo session.*’ (Patient 3-stage I-diagnosed in 2020-PHI)

The majority of PHI policyholders did not mention financial barriers to accessing treatment, nor were they aware that the SHI would allow them to access treatment without having to pay. This is a common misperception that points to a gap in knowledge about their statutory rights. The lack of adequate information about statutory entitlement was also prevalent among those who relied on SHI, and the impact of this misinformation on this group is more severe. Patients with only SHI are at a distinct disadvantage in that they have to make informal payments to continue their treatment. This happens because private hospitals do not comply with social health insurance regulations.

An interview with a participant in the PHI policyholder group (Patient 9-stage II-diagnosed in 2020-PHI) revealed that even patients with PHI can sometimes encounter financial challenges during treatment. The financial challenge she faced was due to the coverage limits on her policy. Despite being insured, she had to pay a significant amount out of pocket for her treatment because the cost exceeded the amount her policy would pay. This is a reminder that the extent of financial protection provided by PHIs varies with individual policy options. The extent of financial protection that PHIs provide also relies on healthcare system factors such as regulations on protections for people with pre-existing conditions and how much leeway private insurance companies have in determining the terms of policy renewal.

All patients in our sample with SHI-only coverage had been asked to make informal payments to receive chemotherapy, radiotherapy, and/or hormone therapy in private hospitals. Non-compliance could result in treatment being denied. Often referred to as ‘physician's visit fees’ or ‘contributions’, these fees were presented as mandatory (but an official receipt for payment was never provided). One patient summarized the payments that were demanded from her during her treatment:

*‘To begin with, there is a physician's visit fee, which I pay once every 3 months. I also pay contributions [referring to extra, informal charges]. I also pay a fee for blood tests before each chemotherapy and a contribution fee that I pay for chemotherapy drugs. This fee varies depending on how much time you spend in a chemotherapy session. For example, let's say you receive 4 hours of chemotherapy, they charge you about 300 TL per session, but if your session lasts between 30 minutes and 1 hour, this fee drops to 200 TL. I don’t know exactly why this is so, and I didn’t ask. I’m still paying contributions for MRI and tomography. … In general, I pay because they say that there is a difference in the procedures performed in private hospitals’.* (Patient 11-stage VI-diagnosed in 2019-SHI)

As exemplified in the above quotation, patients with only SHI have to make several informal payments for treatments in private hospitals. These experiences prevent them from seeing their cancer treatment in private hospitals as their statutory right. As a result, the financial protection function of SHI is essentially non-existent for breast cancer patients.

Insufficient knowledge of the statutory environment is not the only reason SHI patients make informal payments. Some patients had an adequate understanding of their insurance coverage, but they felt that they had no choice but to make these payments in order to access treatment in a timely manner. One patient, for instance, stated:

*‘On the day of my first chemotherapy, they said I’d have to pay 600 TL [one-sixth of the monthly minimum wage at the time of research]. That’s a very large amount, but these drugs are covered by the state, all of them! But we paid, all’s good. No problem with that either. We paid this amount every time. We finished all eight sessions and that procedure was over. Then, they sent me to radiotherapy. I was examined, and they decided how many sessions of radiation I’d need. The subject of payment came up there, too. A large sum was mentioned in the end. … Nothing was free’.* (Patient 2-stage II-diagnosed in 2019-SHI)

The above quotation suggests that awareness of statutory rights does not automatically lead patients to refuse to make informal payments or change their provider. The unequal power dynamics between private hospitals and patients, combined with the perceived and actual problems in public hospitals, leave no room for patients who rely only on SHI to avoid making informal payments to start or continue their treatments in private hospitals.

Ultimately, many patients on SHI had normalised making these informal payments. Taking into account their past experiences and second-hand information from their relatives, they associated public hospitals with long waiting times and delayed treatment, causing them to seek treatment at private hospitals and increasing their willingness to pay. Even patients who had a clear understanding of their insurance and statutory rights were reluctant to take legal action against private providers for fear of being denied treatment.

When SHI ceases to deliver on its financial protection function, patients start economising on healthcare expenditures. For example, one patient faced a difficult decision—whether to pay for a diagnostic service in the hospital where she was receiving treatment or to seek it elsewhere:

*‘They sent me to radiation oncology and asked for a PET scan fee of approximately 15,000 TL. It’s supposed to be free. So we asked why the charge. … Because, they tell you, their device is state-of-the-art. From the moment you enter, you see PET advertisements, posters, and billboards in that department. They had only one PET scan device, so there was no other option. … They tell you about the disadvantages [of other devices]. … When you are concerned about your life, you automatically want the latest technology, but I didn’t think that way this time when I heard 15,000TL. I didn’t want to pay this, so I switched to another hospital.’* (Patient 12-stage II-diagnosed in 2020-SHI)

The above-mentioned quote explains how patients on SHI can be left in a position to make cost calculations for continuing their treatment. This is in stark contrast with the experiences of patients with PHI, as they rarely face such hard choices and are provided with the service without question.

### Service coverage

The treatment pathways of SHI patients and PHI policyholders differ in terms of the reliability of coverage and the services covered. PHI offers reliable coverage, which cannot be said for SHI. Most of the interviewees who had PHI reported no problems accessing comprehensive treatment packages from a single provider. One patient revealed her experience as follows:

*‘Being in the same place makes it easier for physicians to follow your treatment. All the physicians are in communication, and they make decisions together. This put me at ease and made me feel more confident. At least I didn’t let it prey on my mind. Receiving all my treatment in the same place made me feel both safe and psychologically at ease.’* (Patient 10-stage II-diagnosed in 2019-PHI)

As shown above, this patient found it beneficial to receive treatment from a single provider because it made her feel at ease and more confident. The ability to receive treatment from a single provider removes the burden of making provider choices at every stage of diagnosis and treatment.

Several interviewees who had only SHI experienced issues receiving standard breast cancer treatment services from a single provider. They had to shuttle between providers to receive different components of their treatment. The SHI coverage in private hospitals includes only chemotherapy, radiotherapy, and hormone therapy, but not essential screenings like MRI or ultrasound, which is a significant contributor to this issue. One patient who had to shuttle between hospitals described her experience as follows:

*‘This is a very difficult process*. *You’re first in one hospital, and then you’re in another. In the meantime, the assistants forget about your files, and you end up having to send files from one place to another… You actually manage the whole process yourself, as a patient… Each time you have to re-explain who you are.’* (Patient 12-stage II-diagnosed in 2020-SHI)

This quote illustrates the challenges that patients who have only SHI face when they are required to shuttle between hospitals for treatment. Managing the process themselves adds extra responsibilities that become burdensome for patients, a dramatic contrast with the seamless experience reported by PHI policyholders.

Our analysis also sheds light on other significant differences in service coverage. Two patients on PHI (Patient 4-stage III-diagnosed in 2018-PHI and Patient 8-stage III-diagnosed in 2020-PHI) mentioned that they had received information about nutrition, psychological support, and self-care before and after surgery and chemotherapy. This suggests that the care provided by their PHI was more comprehensive. For SHI holders, however, such services fall outside the scope of their insurance.

Notwithstanding their diverse treatment pathways, issues with service coverage appeared at the post-treatment stage for both PHI and SHI holders:

*‘[Referring to the post-treatment stage] One day, when I told the doctor my shoulder hurt, she got very nervous. She said, "I mean, you're an at-risk patient now, so I can't ignore this." She requested an MRI, which cost 2,000 liras, just for a shoulder MRI. You know, I have my annual check-ups in a month, so there will be a whole-body scan. I wonder how much that’s going to cost me.’* (Patient 2-stage II-diagnosed in 2019-SHI)

*‘Treatment is over! What will I do now? Every 3 months, the doctor requests a PET, tests, mammography, an ultrasound, a gynaecological examination… These are extras. Follow-ups after the end of treatment should also be covered.’* (Patient 10-stage II-diagnosed in 2019-PHI)

This shared concern among breast cancer patients highlights the limited coverage of post-treatment follow-up services in both insurance types. The only way for both groups to access free post-treatment follow-up services is to use public hospitals. However, it may be harder for patients to make this provider shift due to their negative perceptions of public hospital services.

### A sense of equitable treatment

The third difference between the breast cancer treatment pathways is that most participants in the SHI-only group expressed a feeling of injustice in their experiences with private hospitals. Both groups referred to actual and perceived disparities between the conditions in public and private hospitals, especially when they explained why they chose to use private services. Patients without PHI, however, also call attention to inequities that arise from the attitudes of private hospitals, which apply different standards for physical facilities during treatment.

One patient reported that even though she was able to access the same medical treatment as a PHI policyholder, the physical conditions were considerably different:

‘*They have a special area on the second floor, an airy place, full of light [which only PHI holders can use]. … Even the rooms are different for people who receive treatment [chemotherapy] through the SHI. There is second-class treatment in the hospital. Let me tell you about the basement floor, B1. There is no natural light, so you [as an SHI holder] receive your chemotherapy in a place with artificial lighting.*’ (Patient 7-stage III-diagnosed in 2020-SHI)

This experience demonstrates an actual disparity in breast cancer treatment pathways in terms of the physical conditions under which patients receive treatment in private hospitals. Although the discrepancy in these conditions does not influence the treatment regimens themselves, patients still felt this difference was important and perceived it as an injustice.

Another SHI holder mentioned a subtle but important disparity she had observed:

‘*If you didn’t get your medical tests done in that [private] hospital, your doctor isn’t able to see your results on his computer screen. Then the doctor forgets to call you the day before the chemo and isn’t able to tell you if you are fit enough to get your chemo, because you didn’t get your tests done in the hospital where they work.*’ (Patient 12-stage II-diagnosed in 2020-SHI)

As discussed in the section on service coverage, it is common practice for patients on SHI to use a combination of different providers during their treatment as a way to compensate for disparities in service coverage between PHI and SHI and to reduce the amount of out-of-pocket payments—getting medical tests done in a public hospital or a private one that offers cheaper prices, for example. In the quote above, the patient reported that using other hospitals to get her medical tests not only makes her responsible for transferring her own medical records between hospitals (also discussed in the section on service coverage), but sometimes it also leads to disruptions in her treatment regimen, even if she submits her medical records on time. These disruptions are possibly a result of carelessness on the part of the hospital staff.

Patients on SHI interviewed in this study were all too familiar with both the SHI’s failure to provide financial protection and its restrictions on service coverage, not to mention the obvious and insidious ways in which they receive differential treatment in private hospitals. These experiences seem to have undermined their confidence in SHI. In addition, most stated that they would like to purchase a PHI policy that would cover their breast cancer treatment, but their cancer diagnosis would make them ineligible. These experiences and conditions may have instilled in some of them a marketised understanding of healthcare.

*‘If you are willing to pay some money, you can get your chemo in a room for two or three people, like me. If you pay a bit more, you can get it in a single room. It’s all about buying comfort. The treatment itself is the same—it’s the same everywhere—but it’s like you have to pay for your comfort.*’ (Patient 6-stage III-diagnosed in 2020-SHI)

The patient quoted above sees out-of-pocket payments as a way to receive treatment comfortably. She describes breast cancer treatment in Türkiye, chemotherapy in particular, as a form of a segmented service-delivery model. In her opinion, the segmentation is not about the treatment regimens; it is about the level of patient comfort during treatment.

## Discussion

We identify two different breast cancer patient pathways based on insurance type in a healthcare system where breast cancer treatment in both public and private sectors is covered by SHI. We present data showing that there are objective and subjective distinctions in treatment pathways. Objective distinctions include the extent of financial protection and the scope and reliability of service coverage that PHI and SHI provide. The subjective distinction is the patient's feeling of being treated equally by private providers. PHI policyholders report easy access to timely and comprehensive treatment. By contrast, the unregulated private hospital sector forces patients who have only SHI to navigate convoluted paths to care, which can include combining providers and calculating costs and affordability.

Our findings partially corroborate recent systematic reviews [7, 8] on healthcare system factors that pose barriers to breast cancer diagnosis and treatment in LMICs. Their results, like ours, indicate cost as a major healthcare system factor that hampers diagnosis and treatment. In Türkiye, however, unlike in LMICs, cost barriers do not lead to complete exclusion from services, because Turkish patients can always switch to public hospitals if they are unable to afford treatment in private hospitals. But the likelihood of them switching to public hospitals is low due to their low level of trust in these providers. Among the patients with only SHI we interviewed, several expressed a deep mistrust of public providers that makes it difficult for them and patients holding similar views to seek treatment at public hospitals. This lack of confidence in the SHI system is, in our opinion, at least partially attributable to shorter consultation times in public hospitals, which is itself a product of policy.

Another finding in our study that mirrors the results of Gbenonsi et al.’s review paper on LMICs [8] is that provider attitudes also obstruct patient access to diagnosis and treatment in Türkiye. However, unlike in LMICs, in Türkiye, it is not a medical staff problem, it is a private hospital problem. Instead of the corruption, bribery and nepotism observed in LMICs [8], the barrier to diagnosis and treatment in Türkiye is that private hospitals request informal payments from patients who have only SHI. The quality of care and the shortages of medical staff, medicines, and technologies observed in LMICs were not mentioned by our participants. This can be explained by the fact that Türkiye is a UMIC and, more specifically, because we focused on the experiences of patients using private hospitals in the country’s most affluent metropolitan city.

This article demonstrates that healthcare system factors lead to bifurcated treatment pathways for breast cancer patients in Türkiye. We identified two major factors that impede access to breast cancer diagnosis and treatment: a failure to fully enforce the mandates of the state’s social health insurance programme in the private hospital sector and a growing reliance on private insurance to gain access to essential services. These two factors are intertwined and together result in a considerable mismatch between patients’ lived experiences and their statutory entitlement to free breast cancer diagnosis and treatment.

First of all, there is a clear manifestation of poor regulation in the private hospital sector: the practice of private hospitals’ requiring patients to make informal payments SHI-covered for standard chemotherapy, radiotherapy, and breast surgery treatment. Patients with only SHI are left in a powerless position vis-à-vis private providers and, as a result, feel compelled to make these informal payments for the unknown duration of their treatment. Patients are often unaware of their statutory entitlements, and they also harbour a distrust of public providers, which increases their reliance on private providers and their willingness to pay. And the considerable stress and anxiety that accompany a breast cancer diagnosis hinder their ability to challenge these unlawful practices.

The second aspect of the Turkish healthcare system that our study brought to light is the increased need of PHI for breast cancer treatment. The increasing importance of PHI for essential services such as breast cancer treatment amongst Turkish patients is striking, which may seem counter-intuitive in a country with universal healthcare that fully covers the cost of breast cancer treatment. Nevertheless, the increased role of PHI in breast cancer treatment is an expected consequence of the failure to fully enforce the mandates of the state’s social health insurance programme in the private hospital sector. Although some patients could switch to public hospitals for all their treatment, many would not choose to do so for a variety of reasons. In addition to mistrust in public hospitals mentioned before, another factor is the increased supply of private hospitals that provide breast cancer care, which has created a significant rise in human and technological resource capacity in the private sector, which will continue to attract patients.

SHI’s failure to provide financial protection and the increased perceived need to have PHI seem to have undermined the ability of SHI patients to choose a provider. Contrary to the Turkish healthcare reform’s promise that patients would be allowed to choose their care provider, breast cancer patients with only SHI have no choice but to shuttle between providers. In addition, echoing Fotaki’s insights [26], our analysis also demonstrates that patients do not always wish to choose a provider at every stage of their diagnosis and treatment. For most patients, choice appears to be important only at the beginning of the treatment process. Once they make this initial choice, they prefer to stay with that provider. However, the ability to receive treatment from a single provider is, in practice, a privilege reserved for patients who have PHI.

This study is not without limitations. One is the self-selection bias. Our participants had more years of formal education than the general population, all having completed high school or higher. Moreover, they all belonged to or knew someone who belonged to a cancer patient organization. We believe these characteristics made our participants more knowledgeable about their diagnosis and better able to articulate their treatment experiences. The participant profile therefore limits our ability to make generalisations about breast cancer patients’ experiences in Istanbul as a whole. Future research would benefit from a focus on less-educated and less socially connected patients. Our exclusive focus on patient experiences in private hospitals is another limitation; we did not include patients who were using public services. Qualitative research on the experiences of breast cancer patients in public hospitals will yield a fuller picture of breast cancer treatment pathways in Türkiye.

## Conclusions

This article underlines the importance of considering healthcare system factors alongside patient-level clinical and demographic factors in identifying and tackling disparities in treatment pathways. Including healthcare system factors will strengthen our frameworks for understanding disparities in treatment pathways and their underlying causes in UMICs with universal healthcare systems. We call for a nuanced, bottom-up approach, combined with a context-sensitive one, to identifying healthcare system factors. This approach will also afford us insights into the mismatches between the statutory characteristics of healthcare systems and patients’ lived experiences. We conclude that healthcare system factors are influential in shaping treatment pathways for breast cancer treatment in UMICs with universal healthcare systems but that the underlying factors differ from the ones in LMICs. Instead of the low quality of healthcare and the shortages of medical staff, medicines, and technologies cited in studies in LMICs, factors in UMICs include the degree of financial protection, the comprehensiveness of coverage, and the ability of the healthcare system to convey a sense of fairness. Last but not least, this article underlines the fact that the influence of healthcare system factors on breast cancer treatment pathways manifests itself not only in the form of granting or denying access to treatment, which is the situation in LMICs, but also by shaping the actual and perceived conditions of access to breast cancer treatment.

## Data Availability

We are unable to make the data publicly available because the ethical approval for this study was granted based on the premise that all data and information regarding participants will be stored in protected hardware with access limited to the authors of this study only. However, the transcripts of interviews in Turkish in an anonymised format and the signed informed consent forms are stored on a secure network drive for a maximum period of 5 years after the collection of the data. Upon request, the deidentified interview data can be accessed by contacting the first author. To fully anonymise the interview data, a unique code was assigned to each interview. These codes can be accessed only by the authors.

## Abbreviations

LMICs: Lower-middle-income countries
PHI: Private health insurance
SHI: Social health insurance
UMICs: Upper-middle-income countries
